# The silent loss of cell physiology hampers marine biosciences

**DOI:** 10.1371/journal.pbio.3001641

**Published:** 2022-05-12

**Authors:** Frank Melzner, Imke Podbielski, Felix C. Mark, Martin Tresguerres

**Affiliations:** 1 GEOMAR Helmholtz Centre for Ocean Research Kiel, Marine Ecology, Kiel, Germany; 2 Alfred Wegener Institute, Helmholtz Centre for Polar and Marine Research, Bremerhaven, Germany; 3 Scripps Institution of Oceanography, University of California San Diego, La Jolla, California, United States of America

## Abstract

The ongoing loss of expertise on the biochemistry and physiology of marine organisms is hampering our understanding of biological mechanisms and will ultimately affect our ability to predict organismal responses to climate change.

## Teaser

An ongoing loss of experts in marine cellular biochemistry and physiology (CBP) is stagnating the generation of knowledge upon which rapidly growing “omics” approaches rely, ultimately hampering our ability to predict organismal responses to climate change.

In the past few decades, the genomic revolution has enabled astonishing advances in marine ecological and evolutionary sciences. Today, genomic and transcriptomic techniques are increasingly being used to attempt to understand and predict the responses of marine species to ongoing climate change—clearly one of the most formidable scientific challenges of our generation. But while researchers were able to identify genomic targets of selection in some recent studies (for example, in coral populations inhabiting exceptionally warm waters [1] and in mussels reared under simulated ocean acidification [2]), the functions of the coded proteins are often poorly characterized or even completely unknown. The few studies in which researchers have successfully linked genomic targets of selection to specific cellular processes (e.g., [3]) share a common feature: They benefited from decades of foundational research on cellular biochemistry and physiology (CBP) that grounded their analysis.

Unfortunately, the genomic revolution has led to the displacement of many disciplines perceived to be less cutting edge, as the number of faculty positions could not keep track with the explosive diversification of biological research. Similar to the widely recognized waning numbers of taxonomists [4], there is a silent and inadvertent loss of CBP experts in the marine science community. Inexorably, this loss in expertise will affect our ability to discover and characterize phenotypes and to predict species-specific resilience and vulnerability to climate change. Meanwhile, biomedical researchers are uncovering a surprising lack of correlation between gene expression and cell function even in clonal cell lines under controlled laboratory conditions [5]. These findings highlight the urgent need for a comprehensive understanding of cellular biological processes that extend beyond genomics and transcriptomics.

**Fig 1:**
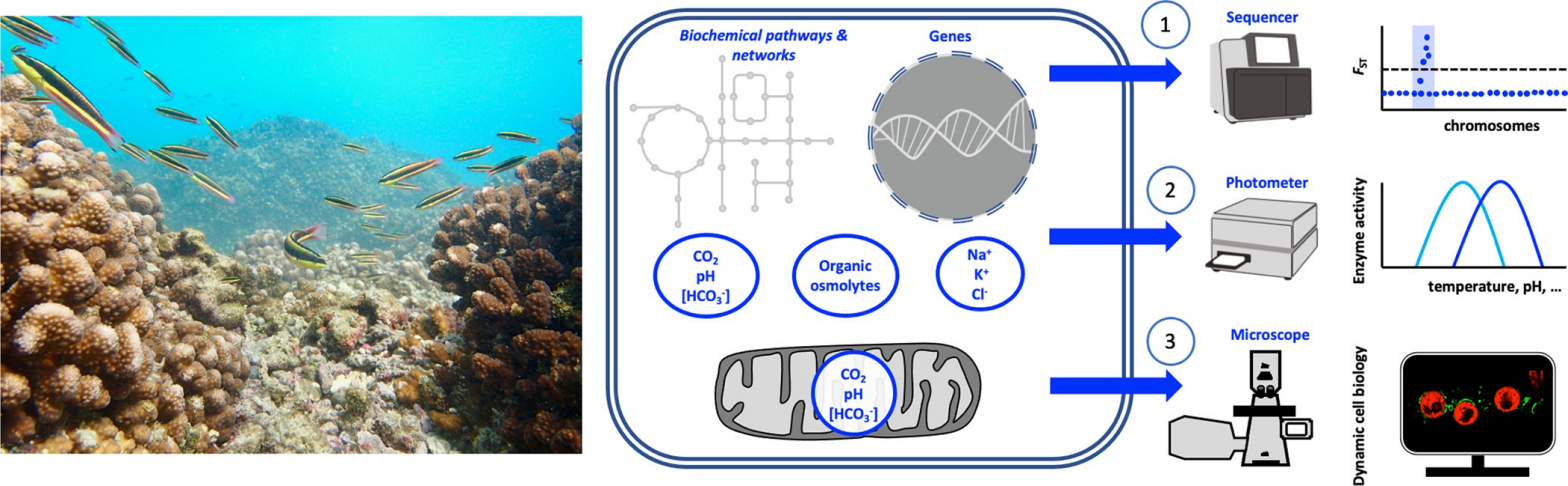
Cooperation between scientists specializing in “omics” and marine CBP is essential for understanding responses and rapid evolution to climate change. Left: Coral reefs are endangered by climate change and harbor thousands of marine species with unique CBP adaptations to their environments (*image credit*: *M*. *Tresguerres*. *Taken at Parque Nacional Cabo Pulmo*, *Baja California Sur*, *Mexico*). Middle: Cells contain complex fluids with defined carbonate chemistry and dissolved organic and inorganic molecules. The roles of many genes, their coded proteins, and their emerging cellular functions are unknown in many marine animals, especially invertebrates. Right: Genome scans (1) can give us information on genetic differentiation (*F*_ST_) of populations selected by climate change-relevant stressors (e.g., temperature or pH). Genomic regions under selection (light blue region) can then be further characterized using CBP methods (shown in 2 and 3). In vitro enzymatic assays (2) can help us understand how mutations (SNPs) in genes under selection can enhance protein function under novel abiotic regimes. These assays require knowledge of cellular fluid composition. Dynamic cell biology approaches (3) can help us elucidate the physiological functions of proteins that are targets of selection (*image credit*: *Angus Thies*, *Tresguerres Lab*). CBP, cellular biochemistry and physiology; SNP, single nucleotide polymorphism.

Cells contain a dense mix of ions, proteins, and other organic molecules (collectively referred to as “osmolytes”) that create a gel-like fluid that determines protein and organelle function (**Fig 1**); the properties of this fluid can greatly vary in manners specific to organelles, cell types, and species [6]. Most metabolic reactions are highly sensitive to carbonate chemistry and osmolyte concentrations, and, virtually, every enzyme is either directly impacted by pH or posttranslationally regulated via acid–base-dependent signaling pathways [7]. Thus, knowledge of intracellular carbonate chemistry under both control and stress conditions is essential for our mechanistic understanding of the impacts of ocean acidification and other stressors. However, although invertebrates constitute approximately 75% of marine animal biodiversity, not one invertebrate species has fully characterized intracellular osmolyte concentrations, acid–base parameters, and carbonate chemistry [6]. Indeed, the most comprehensive (yet incomplete) assessment of intracellular osmolyte budgets in invertebrates was published >60 years ago [8], and detailed information about intracellular acid–base parameters is available only for squid [9]. This lack of knowledge has many implications, such as impairing our ability to mimic intracellular fluids during in vitro characterization of the proteins that are targets of selection (**Fig 1**). Numerous other critical processes are similarly understudied at the biochemical and cellular levels, including aerobic and anaerobic ATP production, antioxidant responses, biomineralization, and metabolic exchange between symbiotic partners. Not coincidentally, a large portion of genes from marine animal pan-genomes cannot be functionally annotated at present.

Of course, “omics” approaches have an important role in marine sciences, both for evaluating known processes and for generating new hypothesis. But do we want to rely on transcriptomics to assess effects of environmental stressors on marine animals when we do not know the identity of the relevant genes and the functions of their coded proteins? How relevant are Gene Ontology or KEGG pathways for studies on marine animals, considering these databases are largely built using model organisms that do not possess the traits we are interested in evaluating? Can we accurately predict the responses of marine fish to climate change thorough studies on zebrafish, a freshwater species? Do rodents or *Caenorhabditis elegans* (or even noncalcifying anemones) hold the key to understanding how corals build massive calcium carbonate reef structures? Similarly, collaborations with traditional molecular biologists and biomedical scientists will certainly help us to gain insights about how marine organisms work [10]; however, this does not eliminate the need for marine CBP scientists who can analyze Big Data in an environmentally relevant context, identify gaps, formulate the next set of hypotheses, and design and execute the ensuing experiments.

The field of marine CBP, alas, has intrinsic limitations that conspire against its progress and impact. Chief among them, method development is very time consuming and often results in protocols that can only be performed in specialized laboratories leading to relatively modest publication and citation rates. In the current hypercompetitive academic environment, this combination can discourage junior researchers and be detrimental to early career faculty. In addition, the small and decreasing size of the scientific community in this field constitutes a major hurdle during the review process. Indeed, it is increasingly difficult to find peers who can fairly assess the quality of work while also avoiding conflicts of interest. As a result, work is often reviewed by colleagues and editors who lack the required expertise and have other scientific interests and thus tend to perceive research on CBP as “too specialized” (or, if reviewed by biomedical scientists, as “not sophisticated enough”). This generates an unfortunate cycle whereby studies are seldomly published in high-profile multidisciplinary journals, affecting the ability of researchers in this field to influence the scientific community and secure funding and faculty positions. Furthermore, the retiring of each colleague results in the loss of an academic role model, completing a “vicious cycle.”

Retirement of colleagues also leads to loss of methodological know-how that, eventually, will have to be redeveloped when cellular phenotyping, in the broadest sense, inevitably becomes essential. In the short term, the theoretical and practical know-how can be preserved through detailed video and written methods and review papers. But in the medium and long term, marine CBP scientists will always be needed to identify original questions, develop new methods, interpret results, and promote and generate excitement to the next generations of scientists and keep the cycle going. We thus urge research institutions to once again value and promote the hiring of marine CBP faculty and researchers. Cluster faculty hires that “pair” them with evolutionary ecologists seem a promising way forward, especially if these researchers work on similar taxa and environments. Only with a strong community of CBP scientists we will be able to understand how marine animal species “work,” how cellular attributes differ from those of biomedical model organisms, and why some genotypes fare better than others in rapidly changing ocean environments.
